# Practical Notes on Diagnosis and Treatment

**Published:** 1911-03-04

**Authors:** 


### Practical Notes on Diagnosis and Treatment.

### Aortic Disease.

Remember that aortic disease is twice as common in the subjects of tabes as it is in the general population.—*Dr. Theodore Thompson.*

### Secretory Activity of the Stomach.

The assistance to be obtained from the repeated examination of the gastric juice as an aid in diagnosis may be summarised as follows: Progressive hypochlorhydria or rapidly developing hypochlorhydria is in favour of carcinoma. Hyperchlorhydria is in favour of functional disease or ulcer, and against cancer.—*Dr. Sidney Martin.*

### Yohimbin.

The influence of yohimbin upon cows has recently been investigated experimentally by Kronacher. The results suggest several useful applications of this remedy in the case of many women's ailments. He found that doses of $\frac{1}{10}$th gramme given in the food morning and evening lead to an increased activity of the udder and an output of considerably more milk than previously. He also found that it is harmless in the early stages of pregnancy, can be tolerated for a long period, and is useful in cases of chronic endometritis, assisting to restore the functions of the uterus.

